# Comparing the effect of gonadotropin-releasing hormone agonist and human chorionic gonadotropin on final oocytes for ovulation triggering among infertile women undergoing intrauterine insemination: An RCT

**Published:** 2017-06

**Authors:** Robabeh Taheripanah, Marzieh Zamaniyan, Atefeh Moridi, Anahita Taheripanah, Narges Malih

**Affiliations:** 1 *Infertility and Reproductive Health Research Center, Shahid Beheshti University of Medical Sciences, Tehran, Iran. *; 2 *Infertility Center, Department of Obstetrics and Gynecology, Mazandaran University of Medical Sciences, Sari, Iran.*; 3 *Diabetes Research Center, Mazandaran University of Medical Sciences, Sari, Iran. *; 4 *Department of Molecular and Cellular Sciences, Faculty of Advanced Sciences and Technology, Pharmaceutical Sciences Branch, Islamic Azad University, Tehran, Iran.*; 5 *Department of Health and Community Medicine, Faculty of Medicine, Shahid Beheshti University of Medical Sciences, Tehran, Iran. *

**Keywords:** Gonadotropin-Releasing Hormone, Chorionic gonadotropin, Insemination, Artificial, Ovulation, Pregnancy

## Abstract

**Background:**

The purpose of triggering in ovulation induction is to induce the final maturation of oocytes and their release from the ovary for fertilization.

**Objective:**

The aim of the present study was to compare the effectiveness of gonadotropin-releasing hormone (GnRH) agonist and human chorionic gonadotropin (HCG) on the final maturation of oocytes and pregnancy rates in intrauterine insemination (IUI) cycles.

**Materials and Methods:**

In this randomized clinical trial, 110 infertile women who were selected for IUI entered the study. Ovulation induction was performed. Group I received 0.1 mg GnRH agonist as triggering and group II received 10,000 IU of HCG. The serum E_2_, LH, and FSH levels were measured at 12 and 36 hr after injection.

**Results:**

LH surge was detected in all patients. LH levels at 12 and 36 hr after triggering was higher in Group I and it washed out earlier than group II (p=0.00). The pregnancy rate was higher in Group I, but the difference was not statistically significant (26.9% vs. 20.8%, respectively p=0.46). Also, the incidence of ovarian hyperstimulation syndrome was not different between the two groups (p=0.11). There was a significant difference regarding the estradiol levels at 36 hours after triggering (p=0.00).

**Conclusion:**

Effects of GnRH on endogenous LH surge is sufficient for oocyte releasing and final follicular maturation. Pregnancy rates and ovarian hyperstimulation syndrome incidence were not different between the groups. We suggest that GnRH agonists might be used as an alternative option instead of HCG in IUI cycles.

## Introduction

Ovulation induction is one of the treatments to reach a higher number of oocytes and to increase the rate of pregnancy. Induction of ovulation during intrauterine insemination (IUI) is associated with higher pregnancy rates especially in unexplained infertility, women with polycystic ovarian disease (PCOD) or in presence of a subfertile man. Final oocyte maturation and ovulation triggering are the most important steps of infertility treatment which depends on mid-cycle LH surge. Although mid-cycle FSH surge also occurs in natural cycles, its effects have been remained unclear. It seems that it has positive effects on the oocyte meiosis resumption. FSH surge promotes the LH receptor in granulosa cells as well as nucleus maturation and also increases the cumulus cells that might be helpful in the luteal phase (1).

Standard protocol for the oocyte maturation and ovulation triggering in stimulated cycles is a bolus dose of HCG, applied when the follicles reach the appropriate size (17-18 mm). A bolus dose of HCG induces ovulation in 34-36 hr, but its significantly longer half-life of more than 24 hr vs. 60 min for endogenous LH surge, is associated with prolonged stimulation of follicles and corpus luteal as well as increases in estradiol secretion and could promote OHSS (2). Also, a high dose of HCG reduces endometrial mitosis and receptivity and may be associated with lower pregnancy rates (3). Other than HCG, GnRH agonists have widely been used in the last two decades to induce ovulation. GnRH agonists induce the LH endogenous surge by their flare-up effect, which is helpful in ovulation triggering. GnRH agonists with shorter half-life better mimic the natural cycles compared to HCG. They may induce enough LH for final maturation of oocytes and ovulation and also induce natural FSH surge that has a lower amplitude compared with LH (4).

A possible advantage of the GnRH-induced LH surge over the HCG-induced LH surge in stimulated cycles for IVF is the simultaneous induction of a surge of FSH resembling the surge in natural cycles. Although the role of the mid-cycle FSH surge in the natural cycle is not fully clear, it has previously been shown to promote LH receptor formation in the luteinizing granulosa cells, nuclear maturation and cumulus expansion (1). Although FSH surge can be induced by a large bolus dose of HCG, it has not been established in randomized clinical trials (5).

GnRH agonists do not have any effect on medium follicular growth due to short LH surge and lack of LH receptor on medium sized follicles, but FSH release may promote the growth of these follicles and release them after 2-3 days. Some studies have revealed that the use of GnRH agonist for oocyte triggering avoids the development of OHSS, even in women at higher risk of OHSS. It seems this mechanism not only increases pregnancy rate regarding tubal patency and mild male factor but also is associated with lower estradiol level and incidence of OHSS (6). The exact mechanism of OHSS prevention is not completely understood. One theory is that the lesser half-life of the endogenous LH surge induced by GnRH agonist, compared with the HCG, induces a shorter and minor secretion of vasoactive elements such as vascular endothelial growth factor (VEGF), which has an important role in the pathophysiology of OHSS (7).

HCG has been used successfully in the final maturation of dominant follicles in assisted reproduction protocols. Due to the prolonged luteotrophic effects of HCG, ovulatory serum concentrations of this hormone may increase the incidence of OHSS (8). A meta-analysis has shown that the use of GnRH or HCG for final oocyte maturation, in which GnRH antagonist was used to inhibit premature LH surge, yields a comparable number of oocytes capable of undergoing fertilization and subsequent embryonic cleavage (9).

In the present study, we aimed to evaluate the pregnancy and OHSS rates in IUI cycles helped with HCG in comparison with GnRH agonist. To our knowledge, our study is the first prospective randomized cross-over study in English literature supporting the hypothesis that GnRH agonist is an effective alternative to HCG for the final oocyte maturation in IUI cycles.

## Materials and methods

**Patients**

**Figure 1:**
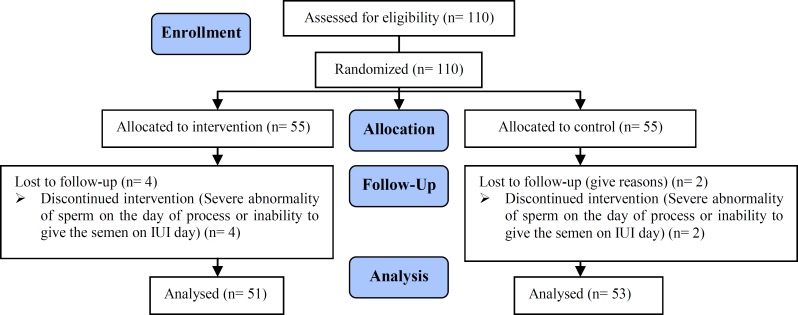
Flow chart of the eligible patients

In this randomized clinical trial, 110 infertile women (range 20-35 yr) who were candidates for IUI entered the study in Imam Hossein Medical Center, Tehran, Iran. Of this, six women were excluded from the study due to a severe abnormality of sperm or inability to give the semen on IUI day. Finally, 53 women were assigned to the intervention and 51 women were included in the control group (Figure 1). 

**Inclusion criteria**

Patients were candidates for IUI who were clomiphene citrate (CC) resistant (patients who do not ovulate while receiving the 150-mg dose of CC), or had less than three follicles on HCG day, or suffered from male sub-fertility or unexplained infertility (10). Other inclusion criteria included normal early follicular FSH, no medication in previous cycles, both tubes patency, the presence of ovaries, and no endometriosis. 

**Exclusion criteria**

Any medical diseases, severe male infertility, poor response ovaries, high response ovary (patients had three or more dominant follicles on HCG day) and any peri-tubal adhesion in hysterosalpingography or laparoscopy. None of the patients had any treatment during the menstrual cycle previous to the stimulated cycle.

**Hyper stimulation protocol**

After an ultrasound scan and estradiol level determination, ovarian stimulation was routinely started on the 3^rd^ day of the cycle. Stimulation consisted of 150 mg clomiphene from day 3-7 of cycle and then a daily subcutaneous injection of 150 IU of recombinant FSH (Gonal F, Serono, Switzerland) from day 8-10 of the cycle. This dose could subsequently be increased or decreased depending on the ovarian response. The follicular monitoring was performed on the 10^th^ day and gonadotropin dose was increased or decreased according to the ovarian response until 1-2 follicles with a diameter more than 18 mm were observed.

The patients were then randomized to receive two different final triggering protocols using a computer based table of random numbers. Four patients in GnRH agonist group (Group I) and two patients in HCG group (Group II) were excluded due to severe abnormality of sperm or inability to give the semen on IUI day. Ovulation triggering was performed by Decapeptyle (Ferring Company, Denmark) 0.1 mg subcutaneously in GnRH group and 10000 unit of HCG (Choragon, Ferring, Denmark) in HCG group (11). Serum levels of FSH and estradiol were measured 12 hr and 36 hr post-ovulation. Samples were obtained and then stored in -70 refrigerators. IUI was performed 36-38 hr later by Walace catheter in the lithotomy position. Finally, about 0.5 ml of semen prepared by swim-up protocol was injected into the uterus.

**Semen preparation and insemination**

Fresh sperm from the partners was used for IUI. All the samples were capacitated using swim-up protocol. IUI was systematically performed 36 hr after the HCG or Decapeptyle injection. 

**Luteal phase support**

Luteal phase support was performed by vaginal progesterone 400 mg (Supp, Cyclogest 400 mg, Actoverco) for 14 days. The chemical and clinical pregnancy rates, and OHSS rates were recorded and compared in two groups. 

**Ethical consideration**

The current study was approved by the institutional ethics committee review board of Shahid Beheshti University of Medical Sciences, Tehran, Iran. All participants gave informed consent before the initiation of the study. 

**Statistical analysis**

All data were analyzed by SPSS software version 22 (Armonk, NY: IBM Corp.). Descriptive data were analyzed by SD and frequency. Linear and logistic regressions were used for correction of the bias. P-value was considered significant if less than 0.05. T-test was used for paired and non-paired quantitative data. Kolmogorov-Smirnov and Wilcoxon tests, as well as contingency tables, were also used depending on the variables and their distribution. The chi-square test was used for independent groups and qualitative data. 

## Results

The mean age (±SD) of the women within the GnRH-a group and the HCG group was 29.4±4.3 and 29±3.9 yr, respectively (p=0.56). Body mass index in two groups were 23.3±3.2 and 23.9±3.9; indicating no statistically significant difference. There was also no significant differences in FSH levels and dose of clomiphene between the 2 groups but the total dose of gonadotropin (rFSH) was higher in group I (p=0.01). The mean number of follicles in right ovary was 5±3.7 in group I vs. 4.5±3 in group II (p=0.45). This number was 4.4±3.3 vs. 4.7±2 in left ovary on the day of HCG injection (p=0.58) (Table I). LH surge was detected in all patients. 

Hormonal values of FSH, LH and estradiol are shown in Table I. GnRH group was associated with the earlier surging of the LH and FSH and also higher levels in circulation compared to the HCG group (p<0.001). Although LH level in HCG group was lower at 36 hr (p<0.001), there was no difference in the FSH level (p=0.87). There was not any significant difference in post-12 hr estradiol level between the two groups, but the 36-hr estradiol level was significantly higher in GnRH group (1230.18±880 compared to 716±692 in the control group) (p<0.001).

Although pregnancy rate was higher in GnRH group (26.9%) in comparison with HCG group (20.8%), the difference was not statistically significant (p=0.46) (Table I). There were two cases of triple and quintuplet pregnancies in GnRH-a group and 2 abortions in HCG group. Two cases of high multiple pregnancies went under embryo reduction in the eighth week of pregnancy, and in both cases, healthy twins were delivered in 38^th^ wk of pregnancy (12). Although the implantation rate in the HCG group (36.53%) was higher than in the GnRH-a group (32.93%), this difference was not statistically significant. Rates of pregnancy, clinical pregnancy, and multiple pregnancy defined as more than one gestational sac on ultrasound during the fifth gestational week were comparable between the two groups.

**Table I:** Demographic data of GnRH-agonist versus HCG in IUI cycles among infertile patients

**Variable**	**Groups**		**p-value**
	**GnRH-a (n=51)**	**HCG (n=53)**	
Mean Age (years)	29.4 ± 4.3	29 ± 3.9	0.58
Mean Clomiphene number	13.7 ± 2.9	13.6 ± 2.2	0.82
Mean rFSH number	8.9 ± 3.7	7.4 ± 1.8	0.01
Mean BMI (Kg/m^2^)	23.3 ± 3.2	23.9 ± 3.9	0.24
Mean FSH base (mIU/ml)	7.5 ± 1.9	6.8 ± 1.5	0.12

Data are presented as Mean ± SD.

Independent t-test was used.

rFSH:

BMI:

FSH:

GnRH-a:HCG:

**Table II:** Hormonal and follicular monitoring of GnRH-agonist versus HCG in IUI cycles among infertile patients

**Variable**	**Group**		**p-value** **
	**GnRH-a (n =51)**	**HCG (n=53)**	
Mean Ro follicles (No)*	5 ± 3.70	4.5 ± 3.00	0.45
Mean Lo follicles (No)*	4.30 ± 3.30	4.7 ± 2.00	0.58
Mean Trigger day (No)*	13.10 ± 1.30	14.40 ± 1.40	0.51
Serum LH 12 hr (mIU/ml)*	64.16±26.95	40.15 ± 13.75	0.00
Serum FSH 12 hr (mIU/ml)*	33.10 ± 11.25	17.99 ± 5.25	0.00
Serum Estradiol 12 hr (pg/ml)*	1119.93 ± 600.25	1206.69 ± 809.77	0.53
Mean Serum LH 36 hr. (mIU/ml)*	11.92 ± 6.01	15.52 ± 6.14	0.00
Mean Serum FSH 36hr (mIU/ml)*	11.07 ± 6.27	11.24 ± 3.88	0.87
Pregnancy Rate (%)*	14 (26.9%)	11 (20.8%)	0.46

LH:

FSH:

GnRH-a:

HCG:

* Data are presented as Mean ± SD.

** Independent t-test.

## Discussion

Based on the results, we found no differences in pregnancy, implantation and fertilization rates between the two triggering agents. Despite our finding that final E_2_ concentrations and the number of retrieved oocytes were higher in the GnRH agonist group, the differences were not statistically significant. OHSS was not observed following the GnRH agonist usage, so even in the same patient population administered the same number of gonadotropin doses; GnRH agonist resulted in lower rates of OHSS without compromising embryo number and qualities. 

Although HCG is widely used for final oocyte maturation, its prolonged luteotrophic effect leads to the development of OHSS. In recent years other drugs such as GnRH agonists have been used for triggering of final maturation and ovulation especially in patients with high risk of OHSS. GnRH agonist has been used for final maturation and ovulation triggering for more than two decades especially as an alternative method for prevention of OHSS. Although it has been recognized that both GnRH agonists and exogenous HCG can induce LH surge, we could show that there are many differences in LH characteristics induced by GnRH agonist versus HCG. We observed that GnRH agonist increases the serum LH level more than HCG in 12 hr post-trigger, but drop earlier than HCG after 36 hr due to a shorter half-life. It seems that these characteristics should be helpful in the prevention of ovarian hyperstimulation syndrome. On the other side, urinary HCG promotes ovarian response and VEGF expression due to a longer half-life and increases the rate of OHSS. 

The flare-up effect caused by a bolus injection of GnRH agonist leads to FSH and LH release and this amount is enough for triggering the LH surge and the final maturation of the oocytes. Preliminary data has indicated that 50 mg of GnRH agonist is the minimal effective dose to trigger ovulation but the best dose is 200-500 mg (13). In our study, we could show that the 0.1 mg of GnRh-a is also effective and LH surge occurred in all patients. Since GnRH does not have any effect on the oocyte quality in IVF or ICSI cycles, the consequent releasing of the medium follicles may increase the pregnancy rate especially in patients with a low response that have no potential risk of OHSS. So we recommend the use of GnRH-an in all IUI cycles for improvement of results. It seems that the flare-up effect of GnRH-a and released FSH and LH (which bind to the corpus luteum receptors and stimulate their hormonal secretion that is needed in luteal phase) might reduce the rate of abortion. As a point, in this study, we did not have any abortion in the GnRH-a group. 

One of the highlights of this study was that the level of FSH before ovulation was higher in Decapeptyl group despite stopping treatment with gonadotropin, which can lead to hormonal reduction. However, the hormone’s physiological increase induced by GnRH agonists and endogenous hormone secretion might have a positive effect on growing and distribution of the cumulus cell masses and thus increase the probability of sperm entering the oocytes (14). Nevertheless, in the GnRH group, an increase was observed on the day after injection. It has been suggested that the physiologic increase in FSH in the pre-ovulation phase of the spontaneous cycle could play a beneficial role in the expansion of the cumulus, thus promoting the passage of the spermatozoa (14, 15). On the other hand, it seems that the conditions closer to the physiological state caused by secretion of endogenous steroid hormones FSH and LH lead to a better balance in steroid levels and thereby an improvement in endometrial receptivity (15).

It is suggested that GnRH-a can prepare better conditions for pregnancy by causing endometrial receptivity without any effect on oocyte/embryo quality as a consequence of a more physiologic endogenous surge of gonadotropins and steroid balance. It seems that GnRH-a can be used in IUI cycles as routine even in patients that are not at risk of OHSS to improve the pregnancy rate. FSH and LH surge occur in the mid-cycle of a physiologic process and are essential for assumption of oocyte meiosis (5). During ovulation triggering using HCG, it binds only to the LH receptor and the FSH effect is omitted and as HCG has a higher lifetime than the natural gonadotropin surge it may provoke the ovarian hyperstimulation.

Unlike HCG triggering of final oocyte maturation, GnRHa triggering is a more physiological approach, eliciting a surge of gonadotropins, similar to that of the natural mid-cycle surge. Thus, in contrast to HCG triggering, GnRHa triggering induces an endogenous surge of FSH as well as LH. Natural dual endogenous LH and FSH surge with GnRH agonists are associated with a shorter half-life and there is no progress to OHSS. On the other hand, luteolysis of corpus luteum is common after GnRH agonist injection due to suppressive effect in the internal LH, especially in assisted reproductive technology. Luteal phase support with progesterone and estradiol overcome this side effect (so luteal phase support is crucial in all GnRH agonist cycles). After the GnRH triggering, a modified luteal phase support including LH-like activity (HCG) or rLH is crucial and the challenge has been to titrate the amount of LH activity needed to sustain the function of only a few corpora luteum after GnRH triggering (16).

Gonen *et al* in a small study used no luteal support following a GnRH-a trigger and obtained three clinical pregnancies. However, in their study CC was used for ovarian stimulation during the follicular phase. Due to the long half-life of CC, a higher pituitary secretion of LH during the luteal phase could be expected to counteract the luteolytic action following the GnRH-a trigger. However, CC is rarely used in controlled ovarian stimulation nowadays (17).

HCG half-life is about 7 days, and it causes changes in the luteal phase and hence increases the VEGF levels in serum and follicular fluid which may be associated with the exacerbation of OHSS (18-20). Small follicles can grow well in the lab conditions, such as the presence of GnRH agonist, which is similar to body physiological conditions. This effect may be associated with an increased chance of multiple births. In the present study, we observed two cases with triple and quintuplet pregnancies in the GnRH agonist group.

Despite some evidence indicating luteal phase defects and miscarriages, we surprisingly had no such findings in the current study; which can be related to the administration of vaginal progesterone. 

## Conclusion

Effects of gonadotropin-releasing hormone agonist on endogenous LH surge are sufficient for oocyte releasing and final follicular maturation. Pregnancy rates and ovarian hyperstimulation syndrome incidence were not different between patients receiving gonadotropin-releasing hormone agonist or human chorionic gonadotropin. We recommend that gonadotropin-releasing hormone agonists could be used as an alternative option instead of human chorionic gonadotropin in IUI cycles. 

## Note

***Registration ID in IRCT: IRCT2015011920408N4***
